# Implementation status of safety measures to prevent errors with non-oncologic methotrexate: surveys in community and hospital pharmacies

**DOI:** 10.1007/s11096-023-01567-z

**Published:** 2023-03-29

**Authors:** Lea D. Brühwiler, Simone J. Gresch, David L. B. Schwappach

**Affiliations:** 1 Swiss Patient Safety Foundation, Zurich, Switzerland; 2 Pharmaceutical Care Research Group, University of Basel, Basel, Switzerland; 3 Institute of Social and Preventive Medicine (ISPM), University of Bern, Bern, Switzerland

**Keywords:** Health services research, High alert drug error, Medication error, Methotrexate, Patient safety, Prevention and control

## Abstract

**Background:**

Accidental overdose of low-dose methotrexate can lead to serious patient harm. Different safety measures are recommended to prevent errors, yet, as errors continue to happen, their implementation is questionable.

**Aim:**

To evaluate the implementation status of safety measures for methotrexate in community and hospital pharmacies.

**Method:**

An electronic questionnaire was sent to head pharmacists of 163 community and 94 hospital pharmacies in Switzerland. The implementation of recommended safety measures (general measures, safety working procedures, IT-based measures) was assessed and descriptive analysis performed. An analysis of sales data underlined the relevance of our results, i.e., the population under risk for overdose.

**Results:**

A response was obtained from 53% (n = 87) of community and 50% (n = 47) of hospital pharmacists. Pharmacies had implemented a median of 6 (IQR 3, community) and 5 (IQR 5, hospital) safety measures overall. Most of these were defined safety procedures, instructing staff on how to handle methotrexate prescriptions. Across all safety measures, compliance with single procedures was perceived as “very likely” by 54% of community pharmacies. IT-based measures (e.g., alerts) were absent in 38% (n = 31) of community and 57% (n = 27) of hospital pharmacies. On average, every community pharmacy dispensed 22 packages annually.

**Conclusion:**

Safety in relation to methotrexate in pharmacies relies mostly on staff instructions, which are considered weak measures. In light of the serious risk imposed on patients, pharmacies should set a focus on stronger IT-based measures that rely less on human performance.

**Supplementary Information:**

The online version contains supplementary material available at 10.1007/s11096-023-01567-z.

## Impact statements

- The study emphasizes that patients using methotrexate are still exposed to preventable risk.
- Many pharmacists have encountered daily instead of weekly accidental methotrexate overdosing. Therefore, pharmacists should be aware of the importance of strong safety measures for error prevention.
- Recommended safety measures are, however, not widely implemented in hospital and community pharmacies making ongoing occurrences of methotrexate overdose likely. With current practices, severe incidents  may not  be prevented.
- Pharmacies should self-assess the type and number of safety measures implemented, so that improvement potential is detected locally.
- Strong, IT-based barriers should be given priority as preventive measures.

## Introduction

Methotrexate (MTX) is used for non-oncologic indications in once weekly regimens [1]. Since once weekly drug regimens are uncommon, patients and health care professionals may easily mistake them for daily regimens [2]. Overdosing can lead to serious patient harm or even death, which is why MTX is considered a high-alert medication [3, 4]. As accidental MTX overdose is at least in theory considered completely preventable, it is defined as a “never event” [5]. MTX tablets are particularly prone to overdosing [6].

The availability of reliable data on error rates is sparse. For instance, in the USA an error rate of 0.4% of MTX packages dispensed was detected by an algorithm [7]. Case reports and pharmacovigilance evaluations show that a significant number of patients continue to be harmed by accidental overdoses worldwide [6, 8–10]. In a recent Swiss analysis, 1–6 patients were annually reported to the national drug authority as having been affected by an accidental MTX overdose [11].

Health care professionals are addressed by international recommendations, e.g., to implement IT-based measures or to adapt local working procedures [1, 12–19]. However, in the last decade, studies have shown that the implementation of safety recommendations in community and hospital care is limited [20–22]. In addition, clinical pharmacist services are—even if proven effective to detect MTX overdoses—not available in all hospitals [23, 24]. In consideration of ongoing overdose reporting, it is questionable whether implementation of recommendations has increased in recent years. This question is particularly important in community pharmacies which dispense the majority of medicines in primary care [25]. Yet also hospitals need to be considered, as transitions between health care settings are prone to accidental MTX overdoses [6].

### Aim

The main aim was to evaluate the implementation status of established safety measures in Swiss community and hospital pharmacies. To assess the relevance of our findings, the second aim of our study was to analyse the amount of MTX sold annually in Switzerland and thus provide an estimate of the population potentially affected by implementation of recommended safety measures or lack thereof.

### Ethics approval

As the study did not include health-related patient data, no approval of an ethical committee was required according to the Swiss federal Human Research Act [26].

## Method

### Survey studies

We conducted a cross-sectional online survey among Swiss community and hospital pharmacies. The questionnaire covered three areas of interest. First, the sociodemographic details of the participating pharmacists and pharmacies were collected. Secondly, participants were questioned on occurrences of accidental MTX overdosing (“have you ever experienced an accidental daily instead of weekly dosage of MTX?”). Participants’ perception about potential harm of overdosing was assessed, using a short medication error description from a published case [2]. Thirdly, it was evaluated which safety measures are implemented in pharmacies, based on international and national recommendations and self-assessment tools [1, 12–19]. We included 17 recommended safety measures for community pharmacies, and 19 for hospitals pharmacies. These were grouped in three categories: (A) general measures (n = 2 for community and n = 3 for hospital); (B) safety measures implemented in the IT-systems (five in point of sales (POS)-software of community pharmacies, and six in computerized physician order entries (CPOE) in hospitals); (C) procedures when handling MTX tablets (n = 10). For the questionnaire, safety measures were phrased as a statement (e.g., “The staff has to be on alert when dispensing MTX” or “The weekday of administration has to be noted as a whole word on the dispensed package”) with three response options («Yes, this is supposed to be done», «No, this is not supposed to be done» and «I don’t/cannot answer the question»). How the safety measures were implemented (e.g., in writing) was irrelevant for these questions. For community pharmacies, we sought further insights, asking participants to estimate how likely it is that the defined procedures are actually followed (4 point Likert scale: very/rather unlikely, rather/very likely). To illustrate this and contextualize the questionnaire items for more realistic answers, a patient vignette was presented: «Miss Koster (32 years) brings a new prescription from her general practitioner for 1 year for «MTX 2.5 mg 20 tablets, dosage: 2-0-0-0» (This dosage is erroneous, it should be 2 tablets once weekly). In the past, your pharmacy has already filled MTX prescriptions by Miss Koster’s general practitioner. She would like to take along a refill.». Items were mostly formulated as closed, single and multiple-choice questions.

The questionnaire was developed based on existing literature and discussions in the research team. Questionnaires were piloted by 13 pharmacists who had partial research experience, worked in the settings, and did not belong directly to the target group. The piloting focused on comprehensibility and technical problems. Based on the feedback, minor adaptions to the questionnaires were made.

### Sample and recruitment

Head pharmacists of Swiss community and hospital pharmacies were eligible to participate.

Community pharmacies were recruited in two regions (i.e., cantons) via their pharmacist associations. With this restriction, we hypothesized that more pharmacists would respond compared to a national survey. Membership in the associations is voluntary, but most pharmacies are members. German speaking cantons with over 50 pharmacies [27] were eligible. So called city cantons were excluded, as they are not representative of other cantons. The cantons of Aargau and St. Gallen were selected, having similar characteristics, but varying in the allowance for physicians to self-dispense to patients directly. With this selection, we reached a sample representative for most Swiss cantons. In January 2022, the two cantonal pharmacist associations sent a non-individualized questionnaire link (EFS survey software, Tivian) to head pharmacists of all 163 member pharmacies. A reminder was sent after 10 days, and the survey closed 14 days after the first invitation. Multiple participation was technically not completely preventable, but would have required active manipulation by participants.

Hospital pharmacies of all general acute care hospitals (n = 105) [28] and hospitals whose head pharmacist was a member of the Swiss hospital pharmacist association (n = 62) were eligible for participation. Duplicates were deleted (n = 52). For nine hospitals, no contact information was accessible. In case that the same pharmacist was listed for multiple hospitals, only the largest hospital was included due to technical reasons (n = 12 excluded). 94 pharmacists received an individualized link to the online questionnaire (Findmind survey software) from the study team, which allowed only one participation in March 2022. A reminder was sent after 11 days, and the survey was closed 20 days after the first invitation.

### Data analysis

Descriptive analysis was conducted. Data were excluded when missing data were noted after the second question. Since missing data were excluded case wise, not all answers for all questions sum to the total sample. Due to rounding, not all answers sum to 100%. No specific action was taken to address item or participant non-response.

The number of implemented measures was analysed once including and once excluding the safety procedure “working attentively”. As this is considered a weak rule from a human factor perspective [29], it is important to keep in mind that including this procedure could have tainted our insight into the implementation of the measures overall.

Means are given with standard deviation (SD) and medians are given with interquartile ranges (IQR). To test associations (between perceived harm and experienced errors, as well as between IT-software and having any IT-based measures), Mann Whitney U tests and Fisher’s Exact test were used as appropriate. Statistical significance was set at 0.05.

### MTX sales analysis

A commercial database (of IQVIA AG, Branch Rotkreuz) was provided by the Swiss Pharmacists’ Association. The database contained sales data of single products from different sellers (pharmaceutical manufacturers, wholesalers, doctors’ suppliers, mail-order pharmacies) to health care providers. The data extract contained the monthly MTX sales to community pharmacies, mail-order pharmacies and to physician offices from 2010 to 2021. The data covered only MTX for non-oncologic usage (ATC-Code L04AX03 introduced in 2009), thus excluding packages of 100 tablets from the database, which were predominantly sold before 2016 when they were retrieved from the market for safety reasons. The extract did not include product names. We analysed the number of single MTX tablets and liquid units (single bottles, prefilled syringes) sold per year.

## Results

### Surveys

87 responses of 163 eligible community pharmacies (53%), and 47 responses of 94 eligible hospital pharmacies (50%) were included in the analysis. Characteristics of participants are presented in Table 1, pharmacy characteristics are given in supplementary tables 1 and 2.

**Table 1:** Characteristics of participants and their corresponding pharmacies

	Community N = 87	Hospital N = 47
Female participants, n (%)	54 (74)	32 (71)
Participants age [median years (interquartile range)]	44.5 (33)	48 (15)
Participants professional experience, n (%)		
0–5 years	17 (22)	6 (13)
> 5–10 years	13 (15)	11 (24)
> 10–20 years	16 (21)	12 (27)
> 20–30 years	21 (27)	14 (31)
> 30 years	12 (15)	2 (4)
Participants position in the pharmacy, n (%)		
Head pharmacist (including pharmacy owner)	60 (76)	30 (70)
Employed	19 (24)	13 (30)
Participants with postgraduate training, n (%)	33 (45)	29 (65)

In both settings, over 90% of pharmacists estimated that MTX overdosing, as described in the case, has a potential for serious or severe patient harm (Table 2). In the community and hospital pharmacies, respectively, 35% (n = 30) and 48% (20) of pharmacists have experienced MTX error by physicians, pharmacists, or nurses (Table 2). Experiences of errors were not associated with the perceived potential for harm (*p* = 0.875 for community and *p* = 0.812 for hospital).

**Table 2:** Perceived potential for harm of accidental MTX overdosing and experienced overdosing errors by pharmacy staff

Perceived potential for harm	Community n (%)	Hospital n (%)
	N = 87	N = 47
No harm	0 (0)	0 (0)
Minor harm	2 (2)	0 (0)
Moderate harm	2 (2)	1 (2)
Serious harm	31 (36)	15 (32)
Severe harm	52 (60)	31 (66)

Experienced prescription error by physician	N = 82	N = 42
Once	20 (24)	11 (26)
Several times	8 (10)	9 (21)
Never	54 (66)	22 (52)

Experienced dispensing error by pharmacy	N = 85	N = 38
Once	6 (7)	5 (13)
Several times	0 (0)	0 (0)
Never	79 (93)	33 (87)

Experienced administration error by nurses		N = 38
Once	N/A	12 (32)
Several times	N/A	0 (0)
Never	N/A	26 (68)

*N/A* Not applicable

### Safety measures overall

Overall, community pharmacies had implemented a median of 6 of 17 evaluated measures (IQR 3, min 0 in 2 pharmacies, max 13 in 3 pharmacies) and hospital pharmacies had a median of 5 of 19 evaluated measures (IQR 5, min 1 in 4 pharmacies, max 14 in 1 pharmacy) (sum of general measures, safety procedures and IT-based measures).

Excluding the “work attentively” rule, community pharmacies had a median of 5.5 measures (IQR 3, min 0 in 2 pharmacies, max 12 in 3 pharmacies). For hospital pharmacies a median of 4 measures (IQR 4, min 1 in 6 pharmacies, max 13 in 1 pharmacy) were in place.

Hospitals, where the pharmacists had experienced errors in the past, had more safety measures implemented, compared to those where the pharmacist had not experienced any error (*p* = 0.003). For community pharmacies, the contrary, but non-significant trend was observed (*p* = 0.689).

### Specific measures

Written standard operating procedures were available in 9% (n = 7) of community and 24% (11) of hospital pharmacies. Patient alert cards were held on stock in 46% (38) of community and 16% (6) of hospital pharmacies. In 58% (11 of 19) of hospitals with a high alert medication list, MTX was listed on it; yet, 26 hospitals did not have such a list.

Community pharmacies had a median of 5 (IQR 2, min 0 in 2 pharmacies, max 10 in 1 pharmacy) safety procedures defined (Table 3). Excluding the “working attentively” rule, 95% (81) of pharmacies had at least one procedure implemented. The relative frequencies of pharmacies, which reported the expected very likely compliance with a specific implemented safety procedure, ranged between 43 and 100% (average 60%, SD 17%).

**Table 3:** Defined safety procedures for MTX implemented in community pharmacies and the reported very likely compliance with the procedure

Procedure	Procedure is defined n (%)	Compliance with the procedure n (% of those having defined the procedure)
Working very attentively	76/83 (92)	60/72 (83)
Dispense only if prescribed by specialist	12/82 (15)	5/11 (45)
Dispense blister product instead of bulk	7/81 (9)	3/7 (43)
Dispense a limited amount	42/83 (51)	24/41 (59)
Max. 1 package	17/83 (20)	11/17 (65)
Max. amount for 4 weeks	3/83 (4)	3/3 (100)
Max. amount for 3 months	22/83 (27)	10/21 (48)
Define weekday together with patient	60/84 (71)	33/58 (57)
Write out weekday in whole word on package	57/83 (69)	42/54 (78)
Teach orally about weekday of intake	68/82 (83)	45/67 (67)
Make sure that intake is known	66/81 (81)	39/63 (62)
Dispense patient alert card	23/79 (29)	10/23 (43)
Document dispensing in specific manner	10/85 (12)	5/10 (50)

The denominator in each question differs depending on the number of responses

In hospital pharmacies, a median of 4 (IQR 4, min 0 in 2 pharmacies, max 10 in 1 pharmacy) procedures were implemented (Table 4). Excluding the “working attentively” rule, 45 (96%) pharmacies had at least one procedure in place.

**Table 4:** Defined safety procedures for MTX implemented in hospital pharmacies

Procedure	Procedure is defined n (%)
Working particularly attentive	25/44 (57)
MTX is not on stock on wards	38/44 (86)
Intake of patients own methotrexate prohibited	26/33 (79)
Special order by wards/physicians	15/46 (33)
Clinical pharmaceutical review of the MTX physician order entry (prescription for the hospital stay)	25/41 (61)
Follow-up of interventions resulting from the review	16/33 (48)
Dispensing MTX in new packaging (not original packaging) to wards	12/42 (29)
Write out weekday in whole word on package	10/41 (24)
Dispense a limited amount	14/41 (34)
Max. 1 (started) package	5/41 (12)
Max. for 1 week	8/41 (20)
Amount adapted to hospital stay	1/41 (2)
Assembly of leftover MTX	12/40 (30)

The denominator in each question differs depending on the number of responses

All community pharmacies stated to have a POS-software. From the hospital pharmacies, 94% (44) have an CPOE. Detailed information on software use is available in supplementary tables 1 and 2.

Implementation of IT-measures is shown in Table 5. Community pharmacies had a median of 1 (IQR 2, min 0 in 31 pharmacies, max 4 in 1 pharmacy) IT-measure implemented. Focusing only on alerts specifically after entry of a daily MTX dosage, 65 of 68 (96%) community pharmacies and 24 of 34 (71%) hospital pharmacies had no such alert (immediate non/interruptive alert or messaging). Hospital pharmacies had a median of 0 IT-measures implemented (IQR 1, min 0 in 27 pharmacies, max 4 in 1 pharmacy).

**Table 5:** IT-based safety measures for MTX implemented in POS-software of community pharmacies and the CPOE-systems of hospitals

	Community n (%)	Hospital n (%)
*After selecting a MTX product in the software*		
Immediate interruptive alert*	25 (34)	4 (12)
Immediate non-interruptive alert*	35 (46)	9 (29)
Informing another person (not the prescriber)	N/A	10 (27)
Yes, a pharmacist		7 (19)
Yes, a nurse		0 (0)
Yes, a physician		1 (3)
Yes, another professional		2 (5)
*After entry of a daily dosage to a selected MTX product*		
Immediate interruptive alert*	2 (3)	1 (3)
Immediate non-interruptive alert*	4 (6)	5 (17)
Informing another person (not the prescriber)	N/A	8 (22)
Yes, a pharmacist		4 (11)
Yes, a nurse		1 (3)
Yes, a physician		0 (0)
Yes, another professional		3 (8)
Default weekly dosage*	29 (40)	N/A

*At least for 1 MTX product. Of those having an IT-based measure implemented, up to 68% of community pharmacists and up to 44% of hospital pharmacists stated that for single IT-based measures the measure is only implemented for some and not for all products. For hospitals however, two of four alerts were implemented for all products. N/A = not applicable

For both community and hospital pharmacies, there was no correlation of having zero or at least one IT-based measure with the type of the software in use (*p* = 0.157 for community and *p* = 0.251 for hospital pharmacies). However, for every POS-software in use, there was at least one pharmacy with at least one measure implemented, indicating that this is technically feasible.

### MTX products

According to the survey, community pharmacies have on average 1.6 (SD 1) and hospital pharmacies have 1.2 (SD 0.45) different MTX products on stock (supplementary table 3). 30% (n = 25) of community and 47% (n = 20) of hospital pharmacies have no MTX on stock. The blister packaged product was the most prevalent product in hospitals and the least prevalent in community pharmacies.

### MTX sales analysis

**Fig. 1:**
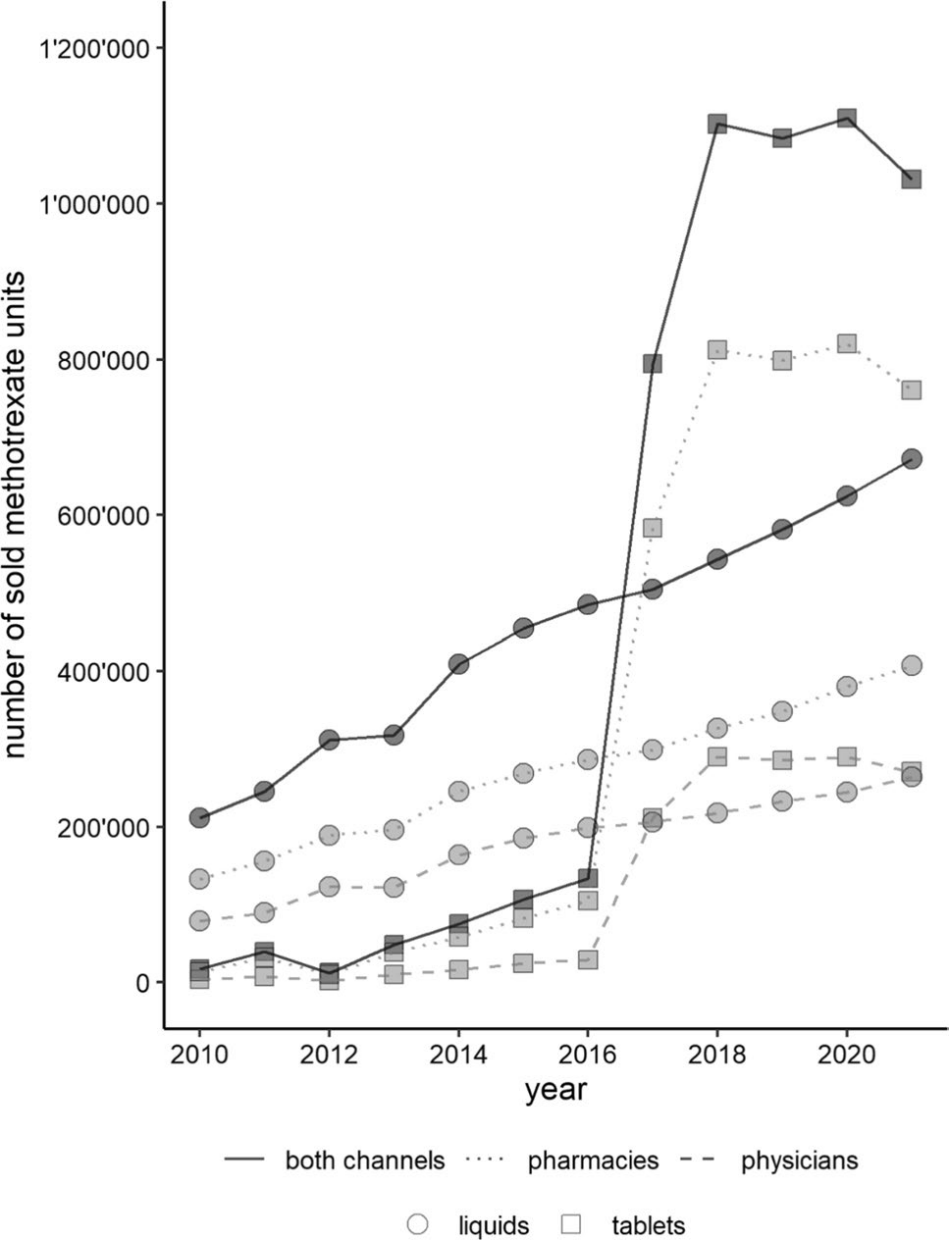
MTX sales per year from 2010 to 2021. Sold units (tablets, liquid units like bottles or prefilled syringes) for non-oncologic use (ATC-Code L04AX03) are displayed by channel (community pharmacies and physicians). This excludes sales of the large package size of 100 tablets which has been abandoned from the Swiss market after 2016. Source: Analysis by Swiss Pharmacists’ Association and Patient Safety Switzerland based on IQVIA sales data

According to the database analysis, the number of sold MTX tablets steadily inclined from 2010 to 2016 (Fig. 1). A major increase was observed around 2016. In the years 2018–2021, stable annually sales of around 1.1 million MTX tablets were registered (around 800′000 to pharmacies and 300′000 to physician offices). On average, calculated for a medium package with 20 tablets, every Swiss pharmacy (n = 1844 [25]) dispensed 22 packages per year. Regarding liquid units, sales increased continuously within the analysed period. Around 671′000 liquid units were sold in 2021. Overall, more tablets and liquid doses were sold to pharmacies than to physician office.

## Discussion

This study assessed the implementation of internationally recommended safety measures in community and hospital pharmacies in Switzerland. Overall, a median of six and five measures were implemented in community and hospital pharmacies, respectively. IT-based measures were completely absent in a substantial number of pharmacies, while procedures in daily work, when handling MTX, were implemented in almost all surveyed pharmacies. Safety procedures are widely established, particularly in community pharmacies, but self-reported compliance varied. MTX is rarely the subject of standard operating procedures or represented on high-alert medication lists. Sales volumes were relatively stable over the last 4 years; around 1.1 million MTX tablets were sold annually, indicating that a considerable number of patients are at risk for accidental MTX overdose.

### Strengths and weaknesses

We surveyed pharmacists on the safety measures implemented in their pharmacy. The self-reported nature of our data must be kept in mind. Our results may overestimate implementation of, and compliance with, safety measures due to several biases.

Bias due to non-responses cannot be assessed, as we lack data on non-respondents. It seems plausible that those interested in patient safety were more likely to participate in the survey, thus potentially limiting the generalizability of our results.

### Interpretation

The MTX sales data indicate that a considerable number of patients are at risk for accidental overdosing and could benefit from rigorous implementation of strong safety measures. With a theoretical average of 22 MTX packages dispensed by a Swiss community pharmacy per year, dispensing MTX are not rare events and thus strong safety measures should be implemented at all pharmacies. Survey responses indicate that pharmacists are aware of the risk of accidental overdose and its serious consequences. Based on these results, one would expect a high level of disposition to install efficient safety measures.

Pharmacies have installed several safety measures, mostly safety procedures describing how to handle MTX. These may be too general considering that accidental overdosing of once daily instead of once weekly MTX being such a specific hazard. Furthermore, the effectiveness of such procedures strongly depends on the individual executing a task. From a human factor perspective, i.e., considering how humans interact with design and technology, such procedures are rated as weak, due to their reliance on human performance [29]. With staff shortages, multitasking or interruptions as well as individuals’ compliance with procedures may deteriorate and lead to errors [30–34]. In fact, self-reported compliance is unsatisfactory in both our and a Dutch study [20]. In the light of the mentioned perspective [29], most recommendations promote rather weak measures, such as clear documentation of the weekday of administration or handing out a patient alert card [1, 13, 15, 16, 20]. The national patient alert card [15] was not available in stock in a substantial number of pharmacies, limiting its effectiveness even more. In hospitals, however, the cards may not be as important, as medicines are only dispensed directly to patients in specific cases.

A more effective recommendation implemented in most hospitals is the quantity limitation of MTX available on wards [13, 16]. This seems easy to follow. In contrast, repackaging as well as quantity dispensing limitations [16] may be disadvantaged by insufficient infrastructure or staffing in smaller hospitals, billing processes in community pharmacies, or by lacking knowledge of safety measures. These measures may therefore show lower implementation. Furthermore, MTX prescription reviews are effective to prevent MTX overdoses [23], but are only done by 61% of hospitals. This may be explained by a generally low emergence of clinical pharmacy services in Switzerland [24].

Regarding IT-based measures, default weekly dosage or hard-stops can very specifically and effectively target the risks of an accidental daily instead of weekly overdosing [29]. They are therefore clearly recommended [13, 17, 19]. However, 38% of community and 57% of hospital pharmacies have no IT-measure implemented at all. This may be caused by the reasonable fear of alert fatigue with unspecific alerts [35] or a lack of knowledge of the technical options in the software. However, every software present in our sample technically offers implementation of at least one measure, indicating that the full safety potential is not yet realized. Additionally, very specific alerts are rare. This is congruent with findings from a study in community pharmacies [21]. Unspecific alerts (after selection of a MTX product) are more often implemented in community pharmacy than in hospital, while it is vice versa for specific alerts.

### Further research

Future research should explore barriers and facilitators for implementation of effective and particularly specific IT-based measures. It could be speculated that the perceived likelihood of accidental overdosing is weighted less important against the anticipated resources required for IT-based safety measures and that it is believed to be compensated for by “being vigilant” working instructions. In a next step, it would be valuable to actively support the implementation of recommendations in pharmacies. This implementation should be accompanied by an in-depth evaluation.

## Conclusion

MTX overdosing errors continue to happen worldwide. Our results indicate that strong safety barriers are rarely implemented, and those measures implemented are unspecific and of questionable effectiveness. The focus of efforts on MTX safety should be on highly effective and specific IT-based measures, like alerts after entry of MTX in a daily dosage. Stronger efforts are needed to prevent additional patients from being harmed due to accidental daily instead of weekly MTX dosing.

## Supplementary Information

Below is the link to the electronic supplementary material.

Supplementary file1 (DOCX 19 KB)
