# Supplementary material for: Implementation status of safety measures to prevent errors with non-oncologic methotrexate: surveys in community and hospital pharmacies
Source: Int J Clin Pharm. 2023 Mar 29;45(3):739–47. doi: 10.1007/s11096-023-01567-z (PMC10250446; doi:10.1007/s11096-023-01567-z)
Supplement: Supplementary file 1 — Supplementary file1 (DOCX 19 KB) [file 11096_2023_1567_MOESM1_ESM.docx]

# Supplementary material

Table 1: further characteristics of community pharmacies

| **Canton, n (%)** |  |
| --- | --- |
| Aargau (47% of 124 pharmacies invited) | 58 (74) |
| St Gallen, Appenzell Inner/Outer-Rhodes (51% of 39 pharmacies invited) | 20 (26) |
| **Pharmacy location, n (%)** |  |
| City centre | 13 (17) |
| Urban quartier | 7 (9) |
| Agglomeration | 15 (20) |
| Countryside | 40 (53) |
| **Chain/Group/Owner, n (%)** |  |
| Group | 34 (46) |
| Private owned | 24 (32) |
| Chain | 16 (22) |
| **Pharmacy with specialisation as “Medinform skin pharmacy”, n (%)** | 14 (19) |
| **Software, n (%)** |  |
| Goldengate (Pharmatic) | 6 (7) |
| Profiline (CSE) | 1 (1) |
| ProPharma X/PPX | 51 (71) |
| Pharmatic Tactil | 14 (17) |
| triapharm (Active) | 10 (12) |

Table 2: further characteristics of hospital pharmacies

| **Type of hospital, n (%)** |  |
| --- | --- |
| University hospital | 4 (9) |
| Cantonal hospital | 13 (28) |
| Regional hospital | 15 (32) |
| Other hospital | 15 (32) |
| **Number of beds** [median beds (IQR)] | 268 (150, 565) |
| **Software, n (%)** |  |
| KISIM | 13 (30) |
| CGM Phoenix (CompuGroup Medical) | 13 (30) |
| inesKIS (ines) | 3 (7) |
| ORBIS (Agfa HealthCare) | 2 (5) |
| Soarian (Cerner) | 1 (2) |
| M-KIS (Meierhofer) | 1 (2) |
| Epic (Epic) | 1 (2) |
| Other | 16 (37) |

Table 3: MTX tablets on stock in the pharmacies. Multiple choice question, 141 answers were given by 84 community pharmacists and 51 answers by 43 hospital pharmacists.

| Methotrexate tablets on stock | Community  n (% of cases) | Hospital  n (% of cases) |
| --- | --- | --- |
| Methotrexate 5 mg bottle 20 pieces | 29 (35) | 5 (12) |
| Methotrexate 10 mg bottle 10 pieces | 27 (32) | 6 (14) |
| Methotrexate 2.5 mg bottle 20 pieces | 19 (23) | 5 (12) |
| Methotrexate 10 mg bottle 10 pieces | 29 (35) | 3 (7) |
| Methotrexate 2.5 mg blister pack 30 pieces | 12 (14) | 12 (28) |
| None | 25 (30) | 20 (47) |
|  |  |  |
|  |  |  |
